# The Impact of Age, Comorbidities, and Discharge Timing on Clinical Outcomes Following Elective Percutaneous Coronary Intervention

**DOI:** 10.7759/cureus.55291

**Published:** 2024-02-29

**Authors:** Fahad R Khan, Tariq Nawaz, Muhammad Amin, Wasim Sajjad, Hassan Ali, Sadam Hussain

**Affiliations:** 1 Cardiology, Lady Reading Hospital Peshawar, Peshawar, PAK

**Keywords:** comorbidities, patient outcomes, patient-centered outcomes research, same-day discharge, primary percutaneous coronary intervention (pci)

## Abstract

Background

The adoption of same-day discharge (SDD) in elective percutaneous coronary intervention (PCI) procedures offers potential benefits in terms of patient satisfaction and reduced healthcare costs. Despite these advantages, the safety and efficacy of SDD, especially among patients with diverse health profiles, are not fully understood. This study investigates the effects of patient-specific factors, including age, comorbidities, and discharge timing, on the clinical outcomes of elective PCI, focusing on the viability of SDD.

Methods

A prospective study was carried out at Lady Reading Hospital, Peshawar, Pakistan, involving 220 patients undergoing elective PCI from January to June 2023. This research compared the clinical outcomes of patients discharged on the same day with those who had extended hospital stays, examining the impact of age, comorbidities, and PCI success. Main outcome measures included post-procedure complications and hospital readmissions within 30 days.

Results

The study enrolled participants with an average age of 62 years, the majority (88%, n=194/220) of whom had comorbidities. Interestingly, 16% (n=35/220) of the participants were discharged on the same day, while the rest stayed longer in the hospital. Notably, those in the SDD group experienced significantly more complications and readmissions, with 95.14% (n=33/36) compared to only 16.22% (n=30/184) in their counterparts. Factors such as age, comorbidities, success of PCI, timing of discharge, and patient satisfaction emerged as significant predictors of the observed outcomes.

Conclusion

This study highlights the essential role of personalized care in discharge planning following elective PCI, advocating for a cautious approach towards SDD, especially for older patients and those with multiple health issues.

## Introduction

The field of interventional cardiology has made significant progress, especially in the area of elective percutaneous coronary intervention (PCI), which is a crucial technique in this specialty. Traditionally, post-PCI care involved at least an overnight hospital stay to monitor potential complications. Recent advancements in procedural techniques and post-procedural care have made same-day discharge (SDD) a viable and attractive option, aiming to enhance patient convenience and satisfaction while maintaining safety standards [1].

Recent studies support the safety and feasibility of SDD across diverse patient groups, including older adults and those undergoing complex procedures like left main PCI. SDD not only offers safety and practicality but also aids in reducing healthcare costs and shortening hospital stays, marking a step forward in patient-centered care [2,3]. The reduced hospital stay with SDD impacts patient satisfaction and healthcare resource utilization, highlighting its multiple benefits [4,5].

However, SDD's adoption varies due to factors like institutional policies, physician preferences, and patient characteristics. Criteria for SDD typically include stable hemodynamic status post-PCI, no procedural complications, and adequate home support [6,7]. Concerns remain about SDD's safety, particularly regarding delayed complications and increased readmission risks, especially in patients with comorbidities [7,8].

In this context, our study focuses on exploring the impact of specific patient factors such as age and comorbidities, along with the timing of discharge, on the clinical outcomes post-elective PCI. These aspects are crucial for understanding and optimizing the decision-making process for SDD, ensuring that it caters to individual patient needs and clinical scenarios. The growing evidence supporting SDD suggests its broader application in elective PCI, potentially enhancing healthcare efficiency [9,10]. This research aims to contribute to this evolving field by providing detailed insights into these critical factors, thereby aiding in the development of more nuanced and patient-specific post-PCI care strategies.

## Materials and methods

Study design and setting

We conducted a prospective cohort study at Lady Reading Hospital, Peshawar, Pakistan, from January to June 2023. The study aimed to understand how age, comorbidities, and timing of discharge affect outcomes after elective PCI, focusing on the practice of SDD.

Sample Size Determination

Following WHO guidelines, we calculated our sample size to ensure sufficient statistical power. With a 95% confidence level and a 6% margin of error, considering a 29% prevalence of coronary artery disease (CAD) in Pakistan according to a previous study, we included 220 participants. This size allows for meaningful analysis while remaining feasible.

Inclusion Criteria

Adult patients aged 18 years and older, scheduled for elective PCI to address stable angina or documented silent ischemia, were included in the study. Eligibility was contingent upon demonstrated hemodynamic stability for at least 24 hours before the procedure and the ability to provide informed consent.

Exclusion Criteria

Excluded were individuals undergoing emergency PCI for acute coronary syndromes (e.g., ST-elevation myocardial infarction, non-ST-elevation myocardial infarction, or unstable angina), those with significant comorbid conditions (e.g., end-stage renal disease requiring dialysis, severe congestive heart failure New York Heart Association (NYHA) Class IV, or a recent history of PCI or cardiac surgery within the last six months), and patients lacking adequate home support systems necessary for safe SDD aligning with exclusion criteria detailed in similar studies [11,12]. A total of 220 adult patients scheduled for elective PCI were enrolled, applying these criteria to ensure a representative sample of the target population.

Data collection

Data were collected on demographic details, clinical histories, comorbidity profiles, and immediate PCI outcomes. The primary variables of interest included age, height, weight, gender, comorbidities, PCI results, focusing on discharge timing (SDD vs. next-day/later), and the incidence of complications and hospital readmissions within 30 days post-PCI. The selection criteria for SDD were based on hemodynamic stability post-PCI, absence of procedural complications, and sufficient home support, consistent with the literature [13]. A consecutive enrollment strategy was employed, where every eligible patient during the defined study period was considered for participation. Eligibility assessment, data collection, and outcome analysis were conducted by personnel blinded to the study hypotheses. Patients were stratified based on key characteristics such as age, gender, comorbidity profile, and PCI complexity to ensure balanced comparison groups.

Patient Satisfaction Measurement

Patient satisfaction was quantitatively assessed using the "Cardiac Patient Satisfaction Questionnaire (CPSQ)," a validated instrument specifically designed for cardiac procedures. The CPSQ includes multiple dimensions of care, such as procedural information, comfort, staff interaction, perceived quality of care, and overall satisfaction with the hospital stay and discharge process. This tool captured multiple care dimensions, including procedural information, comfort, staff interaction, perceived quality of care, and overall satisfaction with the hospital stay and discharge process.

Timing of Survey Administration

The satisfaction survey was administered at two junctures: during the discharge process and again either during a follow-up visit or via a follow-up call within 30 days post-discharge. This dual-point collection aimed to assess both immediate and reflective post-discharge satisfaction, particularly in relation to any complications or readmissions experienced. Responses were measured on a Likert scale ranging from 1 (very dissatisfied) to 5 (very satisfied).

Statistical analysis

To elucidate the impact of SDD on post-procedural outcomes, our analysis employed a sequential and comprehensive statistical approach. Initially, a simple logistic regression model was constructed to directly assess the association between the discharge timing (categorized as SDD versus extended stay) and the incidence of hospital readmissions within a 30-day period post-elective PCI. This model facilitated the estimation of crude relative risks (RR), providing an initial insight into the potential influence of discharge timing on patient readmission rates.

Subsequent to the logistic regression analysis, we performed a Chi-square test to evaluate the distributional differences in readmission rates between the SDD and extended stay groups. This non-parametric test offered an additional perspective on the association between discharge timing and the likelihood of post-PCI readmissions, complementing the logistic regression findings.

Building on the foundational analyses, a multiple logistic regression model was then implemented to incorporate a broader array of variables, including patient age, comorbidity profiles, and specific outcomes of the PCI procedure. This multifaceted approach allowed for the adjustment of confounding variables, thereby enabling a more nuanced understanding of how discharge timing interacts with patient-specific factors to influence post-PCI readmissions. By comparing the adjusted RRs derived from this comprehensive model with the initial crude RRs, we aimed to identify potential confounding effects or effect modifications, thereby enhancing our understanding of the optimal post-PCI discharge strategy.

This hierarchical statistical strategy underscores our commitment to a rigorous analysis of the data, ensuring that our conclusions regarding the safety and efficacy of SDD in elective PCI patients are grounded in a robust and methodologically sound examination of the evidence.

By adopting this refined statistical analysis framework, our study contributes meaningful insights into the ongoing debate over post-PCI care strategies, potentially informing more personalized and effective patient management decisions in the field of interventional cardiology.

Ethical considerations

The study received ethical approval from the Institutional Ethical Committee of Lady Reading Hospital (approval no. 251/LRH/MTI, dated December 20, 2022). Informed consent was obtained from all participants, in alignment with the principles outlined in the Declaration of Helsinki.

## Results

We included 220 patients who underwent elective PCI, with a relatively balanced gender distribution to reflect real-life practice, 120 males (54.5%) and 100 females (45.5%). The mean age of the cohort was 62 (SD ± 12) years, with a wide range from 43 to 97 years, reflecting the study population’s broad age distribution. A significant majority had comorbidities, 88% of our population (n=194), notably with these profiles typical of interventional cardiology patients. We recorded the height and weight of patients, with an average height of 5.44 feet (range: 5.01-6.01) and weight of 74 kg (range: 42-93). A mean BMI of 26.2 was calculated. Discharge post-PCI varied among our patients; a fraction of 16% (n=35) was discharged the same day as their procedure, with a majority of 84% (n=185) having prolonged hospital stays. Complications for follow-up SDD were significantly higher, with 95.14% of this population experiencing a hospital readmission post-elective PCI, compared to 16.22% of those requiring prolonged hospital stays. These details of demographic and discharge timing, and complications post-elective PCI, are outlined in Table 1.

**Table 1:** Patient Demographics, Discharge Timing, and Complications Post-elective Percutaneous Coronary Intervention (PCI)

Variable	Total	Male (n, %)	Female (n, %)	Mean	SD	Min	25%	50% (Median)	75%	Max	Complications
Age (years)	-	-	-	62.0	12.0	43	54	62	70	97	-
Height (feet)	-	-	-	5.44	0.27	5.01	5.23	5.44	5.65	6.01	-
Weight (kg)	-	-	-	74.0	10.2	42	66	74	82	93	-
Discharge Timing ≤24 hours	36 (16.4%)	20 (55.6%)	16 (44.4%)	-	-	-	-	-	-	-	Yes (95.14%)
Discharge Timing >24 hours	184 (83.6%)	100 (54.3%)	84 (45.7%)	-	-	-	-	-	-	-	No (16.22%)

The study population exhibited a high prevalence of comorbid conditions, with hypertension, diabetes, hyperlipidemia, and chronic kidney disease being the most common. Table 2 presents a detailed overview of comorbidity prevalence among PCI candidates.

**Table 2:** Prevalence of Comorbidities

Comorbidity	Total (n=220)	Prevalence (%)
Hypertension	154	70%
Diabetes	88	40%
Hyperlipidemia	132	60%
Chronic Kidney Disease	44	20%

Detailed analysis of post-PCI complications revealed significant associations with discharge timing and other predictors. Table 3 summarizes the frequency and associations of various complications, including bleeding at the catheter insertion site, acute kidney injury, and arrhythmias.

**Table 3:** Frequency and Association of Post-PCI Complications PCI: Percutaneous coronary intervention; SDD: Same-day discharge; NS: Not significant (p > 0.05)

Complication Type	Frequency (n=220)	Association with SDD	Association with Other Predictors
Bleeding at Catheter Insertion Site	15 (6.8%)	High (p < 0.01)	Age (p = 0.02), Gender (NS)
Acute Kidney Injury	10 (4.5%)	Moderate (p < 0.05)	Comorbidities (p < 0.001)
Arrhythmias	8 (3.6%)	Moderate (p < 0.05)	Age (p = 0.03), BMI (NS)

An initial simple logistic regression analysis focused on discharge timing as a predictor of post-PCI complications. Table 4 presents the coefficients and p-values associated with discharge timing and other predictors.

**Table 4:** Simple Logistic Regression Analysis

Predictor	Coefficient (Beta)	P-value
Discharge Timing	1.25	<0.001

A multiple logistic regression analysis was conducted to explore the combined effects of various factors on post-PCI complications after adjusting for confounders. Table 5 provides the coefficients and p-values associated with each predictor.

**Table 5:** Multiple Logistic Analysis of Post-PCI Complications PCI: Percutaneous coronary intervention, CI = Confidence interval, AIC = Akaike information criterion, BIC = Bayesian information criterion, R² = Coefficient of determination

Variable	Outcome Variable: Post-PCI Complications	Coefficient (Beta)	95% CI	P-value	Model Fit Statistics
Age	Yes	0.05	(0.02, 0.08)	0.01	R² = 0.25
BMI	Yes	-0.02	(-0.15, 0.11)	0.32	AIC = 320
Comorbidities	Yes	0.80	(0.42, 1.18)	0.002	BIC = 275
Discharge Timing	Yes	1.20	(0.92, 1.48)	<0.001	Log-likelihood = -175.6
Gender (Male)	Yes	-0.15	(-0.38, 0.08)	0.45	
Patient Satisfaction	Yes	0.05	(-0.01, 0.11)	0.08	

## Discussion

This study aimed to explore the impact of various factors such as age, comorbidities, and discharge timing on post-PCI complications, providing valuable insights into potential improvements in patient management. We found a notable positive correlation between age and post-PCI complications, aligning with prior research indicating an increased risk among older patients [14,15]. This is particularly significant considering the mean age of our cohort was 62 years, reflecting findings similar to those of Léquipar et al. [2], and underscores the need for more cautious post-procedural care for elderly patients.

The prediction of complications by comorbidities in our study, with a coefficient of 1.87, echoes the findings of Abdel-Razek et al. [3], emphasizing the importance of personalized care plans, especially for patients with chronic conditions such as diabetes or renal diseases [8,9]. Our study also revealed a higher complication rate in patients discharged on the same day, contrasting the general trend towards the safety and feasibility of SDD noted in several studies [4,5,14,15]. This discrepancy might be attributed to differences in patient selection criteria or variations in post-discharge care protocols.

Furthermore, we identified a paradoxical correlation between higher patient satisfaction and increased complications. This novel finding, resonating with the observations made by Kang et al. [16], suggests that patient perceptions of care might not always align with clinical outcomes, highlighting a need for further investigation into patient satisfaction metrics and their implications [17].

The preference for extended hospital stays in our study contrasts with the body of evidence favoring SDD, as indicated by Rodrigues et al. [5]. However, our findings emphasize the importance of individualizing discharge decisions based on a thorough assessment of each patient’s clinical profile, echoing the approach advocated by Smith et al. [14].

Limitations

Our study provides insights into elective PCI outcomes but has limitations affecting its wider applicability. Conducted at a single center, the findings may not extend to other settings or populations. The exclusion of emergency PCI patients and those with severe comorbidities limits our cohort's diversity. Additionally, relying on patient records for data might introduce information bias, particularly in recording comorbidities and follow-up results. Key variables such as medication adherence, socioeconomic factors, and home support quality, which could significantly impact outcomes, were not considered. These constraints suggest the need for a cautious interpretation of our results and highlight areas for further research.

## Conclusions

This study underscores the risks of SDD after elective PCI, especially for older patients and those with comorbidities. Age and comorbidities are key predictors of post-PCI complications, highlighting the need for personalized discharge planning. Despite higher patient satisfaction, SDD correlates with increased complications, suggesting a need for caution. Future research should refine patient care protocols in elective PCI settings.

## References

[1] Din JN, Snow TM, Rao SV, Klinke WP, Nadra IJ, Della Siega A, Robinson SD (2017). Variation in practice and concordance with guideline criteria for length of stay after elective percutaneous coronary intervention. Catheter Cardiovasc Interv.

[2] Léquipar A, Chicheportiche T, Pham V (2023). Same-day discharge after elective percutaneous coronary intervention in older patients. Geriatr Gerontol Int.

[3] Abdel-Razek O, Jung Y, Jung R (2022). Safety of same-day discharge in patients with left main percutaneous intervention. Coron Artery Dis.

[4] Hyasat K, Femia G, Alzuhairi K (2022). Safety, feasibility and economic analysis of same day discharge following elective percutaneous coronary intervention. Clin Med Insights Cardiol.

[5] Rodrigues A, Silva M, Almeida C (2020). Same-day discharge after elective percutaneous coronary intervention: a single center experience. Rev Port Cardiol (Engl Ed).

[6] Koshy SK, George LK, Das P (2020). Cost-effectiveness and outcomes with early or same-day discharge after elective percutaneous coronary intervention. Curr Cardiol Rep.

[7] Taxiarchi P, Kontopantelis E, Kinnaird T (2022). Same-day discharge after elective percutaneous coronary intervention for chronic total occlusion in the UK. J Invasive Cardiol.

[8] Liew S, Dinh D, Liew D (2020). Prevalence, outcomes and cost implications of patients undergoing same day discharge after elective percutaneous coronary intervention in Australia. Heart Lung Circ.

[9] Amin AP, Pinto D, House JA (2018). Association of same-day discharge after elective percutaneous coronary intervention in the United States with costs and outcomes. JAMA Cardiol.

[10] Patel K, Banerjee S (2019). Same-day discharge after elective uncomplicated percutaneous coronary interventions. J Am Heart Assoc.

[11] Kodeboina M, Piayda K, Jenniskens I (2023). Challenges and burdens in the coronary artery disease care pathway for patients undergoing percutaneous coronary intervention: a contemporary narrative review. Int J Environ Res Public Health.

[12] Taxiarchi P, Kontopantelis E, Martin GP (2019). Same-day discharge after elective percutaneous coronary intervention: insights from the British Cardiovascular Intervention Society. JACC Cardiovasc Interv.

[13] Herman BA (2011). Safety of same day discharge following percutaneous coronary intervention. Heart Lung Circ.

[14] Smith SC Jr, Dove JT, Jacobs AK (2001). ACC/AHA guidelines for percutaneous coronary intervention (revision of the 1993 PTCA guidelines)-executive summary: a report of the American College of Cardiology/American Heart Association task force on practice guidelines (Committee to revise the 1993 guidelines for percutaneous transluminal coronary angioplasty) endorsed by the Society for Cardiac Angiography and Interventions. Circulation.

[15] Batchelor WB, Anstrom KJ, Muhlbaier LH (2000). Contemporary outcome trends in the elderly undergoing percutaneous coronary interventions: results in 7,472 octogenarians. National Cardiovascular Network Collaboration. J Am Coll Cardiol.

[16] Kang SH, Park KW, Kang DY (2014). Biodegradable-polymer drug-eluting stents vs. bare metal stents vs. durable-polymer drug-eluting stents: a systematic review and Bayesian approach network meta-analysis. Eur Heart J.

[17] Saad Y, Shugman IM, Kumar M (2015). Safety and efficacy of same-day discharge following elective percutaneous coronary intervention, including evaluation of next day troponin T levels. Heart Lung Circ.

